# Helping Haiti’s transportation issues: Increasing Haiti’s medical liabilities

**DOI:** 10.7189/jogh.03.010304

**Published:** 2013-06

**Authors:** Nancy McLaughlin, Hannah Rogers

**Affiliations:** Mercer University, Atlanta, GA, USA

Recently, I went on my first medical mission to Les Cayes, Haiti. I was amazed and astonished at the number of Haitian’s riding motorcycles. However, what was even more astonishing was the lack of safety that was being practiced by the drivers and passengers. This was compounded by the absence of clear traffic laws to govern the use of automobiles and motorcycles alike. I did not realize the gravity of the issue until we got on ground at our medical clinic and started treating patients. One after another, patients arrived with multiple injuries from motorcycle accidents. Some accidents resulted in minor lacerations while others ended with extensive orthopedic injuries and death. After leaving the country, I thought extensively on how this one problem, which greatly impacted the Haitian medical system, could be improved. There are some simple fixes to this issue like providing operators with motorcycle helmets to more extensive remedies such as establishing and enforcing national transportation laws. Nonetheless, I kept coming back to the same question: How did the people of Haiti get to where they are today with an abundance of motorcycles abound and safety measures lagging far behind?

## BACKGROUND

As the poorest country in the western hemisphere and among the poorest in the world, Haiti does not have the infrastructure for a modern working public transportation system [1]. The transportation issues that were present in Haiti only worsened after the horrific earthquake that occurred in Haiti on January12, 2010. The devastating earthquake killed close to 300 000 people and injured hundreds of thousands more. The international community besieged the airport in Port–Au–Prince with equipment to help in the recovery and reconstruction efforts in Haiti. Many roads were so badly damaged that vehicles were unable to navigate them or became damaged themselves as a result. Therefore, many aid agencies began to bring motorcycles into the country to help navigate the treacherous roads. These motorcycles were essential in providing food and medical aid, and in moving people not only throughout Port–Au–Prince and into surrounding villages. Furthermore, organizations such as The Christian Motorcyclists Association distributed motorcycles to pastors so they could provide essential spiritual support to those in hard to access regions [2].

Other aid agencies have similarly utilized motorcycles to distribute medicine and transport patients needing medical care. United States aid agency “Save the Children”, found the country side of Somalia littered with abandoned motorcycles left by earlier aid workers who did not know how to maintain their equipment [3]. The agency paired with Riders for Health to maintain the bikes and train riders to utilize the motorcycles to improve Somalia’s medical system by transporting medicine, patients and health care providers. Riders for Health has expanded its work and implemented similar programs in Uganda, the Gambia, Ghana, Zimbabwe and Nigeria implementing programs in countries with a transportation infrastructure similar to Haiti’s.

As recovery efforts in Haiti have wound down, many of the aid organizations left behind the motorcycles that were so crucial in aid efforts after the earthquake. Abbot, after her first trip back to Haiti after the earthquake, stated that “the contingent that really caught my eye was the army of young males on motorbikes, riding with the cockiness of immaturity exacerbated by the frustration of travel on Haiti's miserable roads.” [4]. Abbot continues [4]:

“Many of them operate as unofficial taxis, transporting customers one by one. In a country near infrastructural collapse, they are an important part of the private sector – and only – transportation system. As part of their scramble to pay for and fuel their bikes, they offer an affordable service that on mountainous Haiti's twisting, gutted roads, is often the only alternative to drudging on foot or mounting the rubbed–raw back of a thirsty, overburdened mule or pony.”

Passengers are not afforded the luxury of motorcycle helmets nor do the driver’s wear any type of protective gear. When speaking to many young men who drive these motorcycles, they almost unanimously stated that they probably would not wear a motorcycle helmet even if given one. This reminds me of a time in the United States when seatbelt laws were being enacted. Many people would not wear the seatbelt, although it had become a law, because it was not “cool” to wear one.

**Figure:**
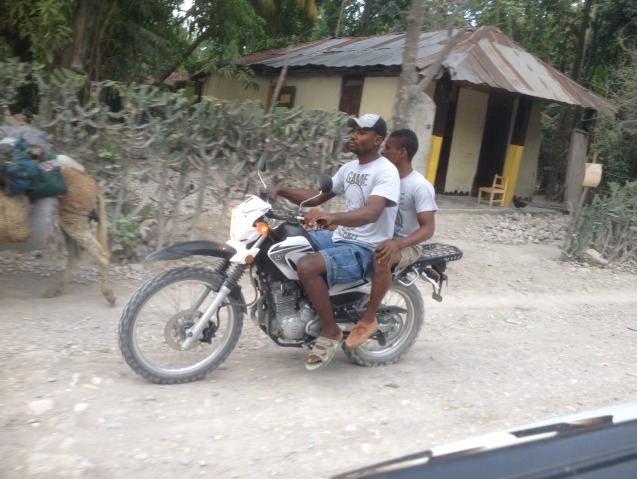
Photo: Courtesy of MaryAnn Reuben

The problem of immature motorcycle operators is only compounded by the abysmal road conditions still present in Haiti, as well as the lack of basic traffic laws. There is no national system to maintain the roadways that are currently present in Haiti. Furthermore, there is limited access to the asphalt needed to repair roadways. Rain, especially during hurricane season, wreaks havoc on the existing roads, making them slippery and muddy, as they are not engineered to facilitate runoff. Traffic lanes are frequently undesignated and populated by pedestrians, livestock, and vendors and guard rails, stop signs, stop lights, and speed limit signs are visibly absent. Drivers often wear headphones to listen to music instead of observing their surroundings and driving defensively, and of course many vehicles are poorly maintained making them a hazard to their operators and others on the road. All of these issues, coupled with immature and reckless operators, cause many otherwise preventable accidents.

## BURDEN ON THE ALREADY STRAINING HEALTHCARE SYSTEM

Haiti’s health care system was poor prior to the earthquake in January 2010, and certainly is in a shambles after it. In Haiti, where the average person makes just over US$ 1 a day, expensive, inaccessible health care can often make the difference between life and death [5]. Even though the Haitian constitution has provisions for universal health care, the reality that health care is easy to access is a farce. Crane et al. note [6]:

“Forty percent of the population lacked access to care, particularly in rural areas of the country. Lack of access has two dimensions: lack of physical access, as 13 percent of the population lives more than 15 kilometers away from the nearest health center, and lack of financial access, as cost–recovery policies in place in most institutions require fees for services that are unaffordable to a large proportion of the population.”

Conditions are rarely treated in a timely manner, which only compounds their severity and increases the morbidity and mortality rates.

Even if Haitians could readily access facilities and had the financial means to pay for care, they would have to face the challenges of finding qualified physicians and nurses. While current numbers for physician density in Haiti are not generally available, in 2001 Haiti had only three physicians for every 10 000 inhabitants; most were concentrated in the capital [6]. In addition, the physical facilities of hospital are dilapidated and experience frequent power outages and water shortages. The equipment utilized to deliver medical care is usually outdated and/or broken. Furthermore, medical facilities often lack the administrative support staff to deliver quality care to those in need.

Haiti’s major health problems are similar to those of other third world countries: low immunization rates, high prevalence of vector borne diseases, HIV/AIDS, and high infant mortality rates. These medical conditions all pose a significant strain on the medical assets that Haiti does have to offer. Trauma–related motorcycle accidents are a major issue that is poorly reported by scholarly literature. It only takes a few days on ground in Haiti, providing medical aid to begin to realize that motorcycle accidents tax an already fragile health care system.

## CASE STUDY

A 35-year old Haitian male presented to the city hospital’s emergency department on Wednesday with an open unstable tibia fracture after being hit as a pedestrian by a motorcycle operator who was driving carelessly. He was treated with pain medication, a long leg posterior splint, given a prescription for antibiotics and analgesics and told to return to the hospital courtyard near the operating room the next day to see if he would be able to have surgery. He was unable to purchase both the analgesics and the antibiotics; therefore, he chose the analgesics. He was told to return to the hospital courtyard near the operating room the next day to see if he would be able to have surgery. He waited all day Thursday but was unable to have surgery because an orthopedic surgeon was not available. He returned on Friday and once again waited but was unable to get surgery. At this time, blood had soaked his dressings and there was no projected date at which he would be able to undergo surgery.

In comparison, this same injury would have been treated in a dramatically different way in the United States. Open fractures often become contaminated; therefore great diligence is paid to prevent infections which are preempted by devitalized bone and soft tissue. The main treatment goals are: limb salvage, prevention of infection, and restoration of function. Therefore, open fractures also require administration of tetanus prophylaxis and intravenous antibiotics with a cephalosporin, such as cefazolin, which would require an inpatient admission [7]. Consequently, a fracture such as this would typically be taken to the operating room very early after the incident and an intramedullary nailing would be performed to stabilize the fracture. Delay in the treatment could result in osteomyelitis, amputation, or even death.

## CONCLUSION

In a case like Haiti an ounce of prevention is worth a pound of cure. Educating inexperienced motorcyclist will be the challenge. Motorcycle safety is undoubtedly one of the last things that cross a driver’s mind until he is involved in an accident. Because of infrastructural limitations, the actual burden placed on the Haitian medical system by these medical emergencies is hard to estimate, but anecdotally any health care provider who has spent time in Haiti will tell you that this is a major issue. Without governmental intervention and enforcement of traffic and safety laws, there is no real way to decrease the number of trauma related to motorcycle accidents.

Other developing countries face the same problems as Haiti. Iamtrakul et al. completed an extensive analysis of traffic accidents involving motorcycles in Thailand and the outcomes of the patients [8]. Their work showed the following results: 1) men had twice as many motorcycle accidents as women, 2) the severity of men’s over women’s injuries were twice as great, 3) accident severity of drunk drivers was four times higher than normal drivers, and 4) drivers not wearing helmets had 6 times greater severity of injuries over those patients who were wearing helmets at the time of their accident. A Nigerian study showed that motorcycles are often useful for navigating poor roads and traffic hold–ups, but the riders often ignore safety measures, making them more vulnerable to accidents [9].

Governmental intervention would have to occur on multiple levels. Instituting just a few of the following prevention strategies would greatly decrease the burden on the health care system. First, riders need to be educated about personal protective gear that could be worn to help prevent injury. Obviously, a mandatory helmet law would be a huge step in the right direction. Solagberu et al. [9] noted that studies have shown that “limb and head injuries are the most common causes of morbidity and mortality in motorcycle accidents, attributing the latter to low use of crash helmets in Nigeria, a situation seen in other developing countries.” This illustrates that the issue of helmet use is not unique to Haiti. Road rules and traffic laws need to be instituted to cut down on the confusion that drivers experience while negotiating Haiti’s treacherous roads. Johnson and Adebayo [10] were able to show that basic motorcycle safety classes significantly decreased the morbidity and mortality among Nigerian motorcycle riders. Iamtrakul et al. [8] recommended graduated licensing to combat the issue of young drivers in Thailand. Limiting the number of passengers allowed to ride on a motorcycle at any one time would reduce the potential number of injuries. Basic road maintenance should be performed to help fill potholes and clear debris from the street. Finally, requiring yearly safety inspections of motorcycles would also be useful.

All of the previously mentioned interventions would help to decrease the morbidity and mortality rate that Haiti experiences from the vast number of motorcycle accidents that occur daily. Most of the mentioned interventions require a strong governmental presence, which is currently a challenge to a politically unstable Haiti. In the meantime, the associated trauma that occurs as a result of these motorcycle accidents will only continue to drain the medical assets of an already struggling medical system.
